# An Empathy-Driven, Conversational Artificial Intelligence Agent (Wysa) for Digital Mental Well-Being: Real-World Data Evaluation Mixed-Methods Study

**DOI:** 10.2196/12106

**Published:** 2018-11-23

**Authors:** Becky Inkster, Shubhankar Sarda, Vinod Subramanian

**Affiliations:** 1 School of Clinical Medicine Department of Psychiatry University of Cambridge Cambridge United Kingdom; 2 Wysa London United Kingdom; 3 Wysa Bangalore India

**Keywords:** mental health, conversational agents, artificial intelligence, chatbots, coping skills, resilience, psychological, depression, mHealth, emotions, empathy

## Abstract

**Background:**

A World Health Organization 2017 report stated that major depression affects almost 5% of the human population. Major depression is associated with impaired psychosocial functioning and reduced quality of life. Challenges such as shortage of mental health personnel, long waiting times, perceived stigma, and lower government spends pose barriers to the alleviation of mental health problems. Face-to-face psychotherapy alone provides only point-in-time support and cannot scale quickly enough to address this growing global public health challenge. Artificial intelligence (AI)-enabled, empathetic, and evidence-driven conversational mobile app technologies could play an active role in filling this gap by increasing adoption and enabling reach. Although such a technology can help manage these barriers, they should never replace time with a health care professional for more severe mental health problems. However, app technologies could act as a supplementary or intermediate support system. Mobile mental well-being apps need to uphold privacy and foster both short- and long-term positive outcomes.

**Objective:**

This study aimed to present a preliminary real-world data evaluation of the effectiveness and engagement levels of an AI-enabled, empathetic, text-based conversational mobile mental well-being app, Wysa, on users with self-reported symptoms of depression.

**Methods:**

In the study, a group of anonymous global users were observed who voluntarily installed the Wysa app, engaged in text-based messaging, and self-reported symptoms of depression using the Patient Health Questionnaire-9. On the basis of the extent of app usage on and between 2 consecutive screening time points, 2 distinct groups of users (*high users* and *low users*) emerged. The study used mixed-methods approach to evaluate the impact and engagement levels among these users. The quantitative analysis measured the app impact by comparing the average improvement in symptoms of depression between high and low users. The qualitative analysis measured the app engagement and experience by analyzing in-app user feedback and evaluated the performance of a machine learning classifier to detect user objections during conversations.

**Results:**

The average mood improvement (ie, difference in pre- and post-self-reported depression scores) between the groups (ie, high vs low users; n=108 and n=21, respectively) revealed that the high users group had significantly higher average improvement (mean 5.84 [SD 6.66]) compared with the low users group (mean 3.52 [SD 6.15]); Mann-Whitney *P*=.03 and with a moderate effect size of 0.63. Moreover, 67.7% of user-provided feedback responses found the app experience helpful and encouraging.

**Conclusions:**

The real-world data evaluation findings on the effectiveness and engagement levels of Wysa app on users with self-reported symptoms of depression show promise. However, further work is required to validate these initial findings in much larger samples and across longer periods.

## Introduction

### Background

Major depression is a disabling disorder with symptoms such as feelings of sadness, worthlessness, and losing interest in activities. Depression is the single largest contributor to global disability with an estimated 300 million or approximately 4.4% of the world’s population (2015) affected by it [1]. Severe depression can lead to suicide, which was the second leading cause of death among people aged 15 to 29 years globally in 2015 [1]. Major depression has been found to impair quality of life [2] and psychosocial functioning [3,4], which is a person’s ability to perform daily activities and to maintain interpersonal relationships.

The economic burden of depression is rising. The cost of major depression in the United States was estimated at US $210.5 billion per year in 2010, an increase of 21.5% from 2005 [5]. For every dollar spent treating major depression in 2010, US $4.70 was spent on direct cost of related illnesses, and an additional US $1.90 was spent on reduced workplace productivity and costs associated with suicide linked to depression [5]. According to the Centre for Mental Health policy paper (2010), the total cost of mental ill health in England was estimated at £105.2 billion a year from 2009 to 2010, an increase of 36% from 2002 to 2003 [6]. The Farmer-Stevenson review that was launched by the UK Parliament in 2017 on mental health in the workplace placed the cost to employers due to poor mental health at £33 to £42 billion a year, with over half of it coming from presenteeism [7]. According to the World Health Organization (WHO) Mental Health Atlas 2017, government spend globally on mental health in 2015 was less than 2% of the global median of government’s health expenditures overall, which has only exacerbated the situation [8].

Mood disorders can be treated by pharmacotherapy or psychotherapy [9]; however, significant treatment barriers remain, such as major shortage of mental health professionals, long waiting lists for treatment, and stigma. The WHO Mental Health Atlas 2017 reported that there is a global median of 9 mental health workers including approximately 1 psychiatrist per 100,000 people [8]. In India, there are approximately 10 mental health professionals for 100,000 people affected by mental health problems [10]. According to the Impact Assessment report from the UK Department of Health (October 2014), access to services for people with mental health problems is more restricted, and waiting times are longer than for other health care services [11]. A 2018 British Medical Association research briefing stated that two-thirds of the National Health Service (NHS) mental health trusts in the United Kingdom had year-long waiting periods before therapy started, and in some locations, waiting periods were close to 2 years [12]. Perceived public stigma, a known barrier, is the degree to which the general public holds negative views and discriminates against a specific group. Young adults who reported higher scores on the Patient Health Questionnaire-2 (PHQ-2) showed greater associations with perceived public stigma than personal stigma [13]. The WHO World Mental Health Surveys show that apart from perceived stigma, structural barriers such as finance and lack of service availability were the most reported barriers to treatment among those with severe disorders [14].

### Prior Work

Face-to-face therapy and guided self-help techniques such as cognitive behavioral therapy (CBT) and behavioral activation are known to be effective in treating depression [15,16]. Face-to-face therapy only provides point-in-time support and cannot scale quickly to address growing mental health challenges. Innovative delivery methods are required to supplement care. Studies have shown that certain user groups are opening up to technology about their mental health problems. A recent study showed that participants reported more posttraumatic stress disorder symptoms when asked by a virtual human interviewer compared with a gold standard assessment [17]. Guided internet-based self-help interventions have been observed to have positive effects on patients with symptoms of depression and to reduce risk of symptom deterioration [18-22]. Mobile app–administered therapy either stand-alone or in blended mode has been found to show positive effects on patients with depression across severity levels in randomized controlled trial (RCT) studies [23-28]. However, there are studies with mixed findings about the benefits of smartphone or online-administered interventions. A recent RCT study that examined the effects of an online mindfulness meditation app compared with an active sham meditation control app found that mindfulness improved across university student participants in both groups, and there seemed no added benefit from offering progressive and varied mindfulness tools [29].

Text-based messaging (internet or smartphone) either with a human coach or with a machine (chatbots) has found increasing adoption in recent years. Artificial intelligence (AI) text-based conversational agents have the ability to offer contextual and always-available support. Studies using internet-based, one-to-one text-based chat interventions for psychological support have shown feasibility and positive improvement in mental health outcomes when compared with wait-list conditions [30]. Two recent studies measured the efficacy of a fully automated mobile conversational agent in the delivery of mental well-being [31,32]. Our study aims to add to the research and evidence base on the effectiveness and engagement levels of AI-enabled, text-based, conversational mobile mental well-being apps.

### Wysa, a Smartphone-Based Empathetic Artificial Intelligence Chatbot App for Mental Well-Being

Wysa, developed by Touchkin, is an AI-based *emotionally intelligent* mobile chatbot app aimed at building mental resilience and promoting mental well-being using a text-based conversational interface. The Wysa app assists users to develop positive self-expression by using AI to create an external and responsive self-reflection environment. Engaging with the app is free and available 24×7, but accessing a human coach via the app is a paid service. We used an early in-the-market app version (see Multimedia Appendix 1) that included only the free always-available chatbot service (not the paid coach service). The app responds to emotions that a user expresses over written conversations and, in its conversation, uses evidence-based self-help practices such as CBT, dialectical behavior therapy, motivational interviewing, positive behavior support, behavioral reinforcement, mindfulness, and guided microactions and tools to encourage users to build emotional resilience skills. The Wysa scientific advisory board approves all content and tools. The conversation-based tools and techniques encourage users to manage their anxiety, energy, focus, sleep, relaxation, loss, worries, conflicts, and other situations.

The app can be downloaded from the Google Play Store and from the Apple App Store. There is no user registration to sign in and no personal identifiable information is asked at any time during app use. Wysa was described as “friendly” and “easy to use” in a youth user study conducted by Wellcome Trust, United Kingdom, Neuroscience, Ethics, and Society Young People’s Advisory Group at the University of Oxford, and BBC Tomorrow's World [33]. The app was adapted and implemented at Columbia University’s SAFE Lab as a tool to provide support to at-risk communities in inner cities (Brooklyn and Chicago), many of whom are gang-involved youth. Although, Wysa is not a medical device, when used as a health and well-being support tool, it can support clinical services as seen from its use at the NHS North East London Foundation Trust [34].

### Study Objective

The primary study objective was to determine the effectiveness of delivering positive psychology and mental well-being techniques in a text-based conversational mode using the Wysa app on users with self-reported symptoms of depression. Users were presented with the validated Patient Health Questionnaire (PHQ-9) during their conversations and screened for selection based on their 2-item (PHQ-2) score. The average improvement in self-reported symptoms of depression (Pre-PHQ-9 minus Post-PHQ-9) was compared between 2 comparison groups: (1) more engaged app users (“high users” group) and (2) less engaged app users (“low users” group).

Our secondary study objective was to understand users’ in-app experiences during app use. A qualitative thematic analysis, as proposed by Braun and Clarke, 2006 [35,36], on in-app feedback responses was performed.

## Methods

### Ethics

The study involved a remotely screened, anonymous nonclinical global population (ie, real-world *in-the-wild* data) and was, therefore, exempt from registration in a public trials registry. The users downloaded the app after having agreed to the Wysa app Terms of Service and Privacy Policy, which included consent to use anonymized data for research purposes. Minimal deidentified data required for the study were used. For details on app specific ethical practices, see Multimedia Appendix 2 *.*

### Study Design

**Figure 1:**
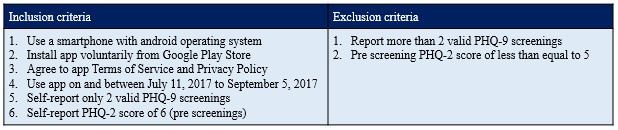
The study inclusion criteria. PHQ: Patient Health Questionnaire.

The Wysa app was downloaded from the Google Play Store voluntarily by geographically dispersed users. The users were filtered for eligibility from a pool of anonymous Wysa app users based on the inclusion criteria (see Figure 1). For the study, we solely looked at user-provided data that were collected by the app during active use. Given the anonymity and nonavailability of user profiles, qualitative and quantitative data were collected concurrently during the study period on and between July 11, 2017, and Sept 5, 2017. These data consisted of user responses to the app’s inbuilt assessment questionnaire and responses to the app-designed text-based conversations and questions. No additional research-framed questionnaires or user feedback questions were designed or issued for repeated interval data collection.

On the basis of the extent of app usage on and between 2 consecutive PHQ-9 screenings, 2 comparison groups emerged (“high users” and “low users”). The users in both groups voluntarily reported 2 valid time point PHQ-9 scores: one at onboarding (first assessment, “Pre-PHQ-9”) and the other on or after 2 weeks (second assessment, “Post-PHQ-9”). The 2 screening time points were considered valid if during the study period only 2 surveys were responded to within a gap of 14 or more days. The “high users” consisted of users who engaged with the app on the 2 screening days as well as at least once between those days. The “low users” consisted of users who only engaged on the 2 screening days but never between those days.

The authors decided to implement a quasi-experimental (simple pre-post) mixed-methods approach given our study objective and the nature of the data being collected. For details on the mixed-methods design and approach, see Multimedia Appendices 2 and 3 *.* See the study recruitment flow diagram in Multimedia Appendix 4.

### Quantitative Measurement and Screening

The inbuilt app-administered assessment questionnaire (PHQ-9) required users to recollect problems over the last 2 weeks; notably, this form of data collection is neither momentary nor *real-time* capture. For details about PHQ-9, see Multimedia Appendix 2. The PHQ-2 score was generated from responses to the first 2 items of the PHQ-9 (ie, range: 0-6). The PHQ-2 is intended for use as an initial screening of depression symptoms, whereas the PHQ-9 score is then used for monitoring depression symptoms [37]. As the app engaged with anonymous users, there was no information available about clinical history and diagnosis. Remote digital screening for depressive symptoms in anonymous populations is very challenging in the absence of face-to-face clinical interviews; therefore, we selected the most stringent threshold based on recommendations in the scientific literature [37], which required a PHQ-2 score of 6.

### Data Collection and Analysis

The app takes the user through conversational pathways based on a user’s interaction. This path varies for every user, based on their messages and context. At various points in a user’s conversational journey, a user is presented with app-designed open- and closed-ended questions that check the helpfulness of these sessions and seeks user feedback (in-app feedback; eg, at the end of every wellness session or at end of every mindfulness or physical activity tool-based session). This voluntary feedback provided by the users was not scheduled repeatedly nor was it used to measure changes in behavior or emotions of an individual over time. Instead, the objective was to understand the users’ experiences and engagement with the app. For the in-app feedback questions, see Multimedia Appendix 5. All transmissions to and from the app were encrypted using recognized security standards and were securely stored in a private cloud server. All user-generated conversations and screening responses were checked for compromise (eg, malicious bots) and deidentified for app identifiers. At onboarding, the following user context information was collected:

1. Major event or recent changes: The response to the question, “What has been the major event or change in your life recently?” was collected by the app in free-text before a Pre-PHQ-9 screening.
2. Ability to cope with daily tasks: Immediately after the Pre-PHQ-9 screening, based on the score, users were asked about their ability to cope with daily tasks. For high severity PHQ-9 scores, users were asked “Is it getting hard for you to cope with your daily tasks?,” whereas for none to mild severity, they were asked “Are you happy with how life is going at the moment?” The user could respond either by clicking preformatted options or by free-text.

For a typical user app engagement, see Multimedia Appendix 6 *.* Microsoft Excel software was used for data wrangling and analysis. Open-source python software on Jupyter Notebook was used for machine learning (ML) modeling.

### Quantitative Analysis Method

#### Impact (Pre-Post) Analysis

To quantify the app impact, the average improvement (pre-PHQ-9 minus post-PHQ-9) was compared between the 2 user groups. A Mann-Whitney *U* test was carried out to test the hypothesis that high users would have greater average improvement than low users. The effect size was measured using the nonparametric common language effect size (CL), calculated as [1-(U/n_h*_ n_l_)], where U was the Mann-Whitney *U* and n_h_ and n_l_ are the numbers of high users and low users, respectively [38]. The CL gives the probability that a user picked at random from the high users group will have a higher average improvement than a user picked at random from the low users group [38,39].

#### Context/Descriptive Analysis

To maintain user anonymity, the app did not capture personal identifiable information or sociodemographic information (except time zone). To capture useful context about users, an analysis of the qualitative responses to key app-based questions was performed, including days of active use, recent major event or changes, ability to cope with daily tasks, and completion of wellness tools.

### Qualitative Analysis Method

#### Engagement Effectiveness

An analysis of users’ in-app feedback responses was performed using thematic analysis [35,36] to measure engagement effectiveness. Main themes and subthemes, derived from the analysis, helped understand users’ app experience and engagement. Prevalence of a theme was measured based on count of response instances and number of responding users. Further insights were identified by intersecting derived user context with the main themes. For details on thematic analysis approach, see Multimedia Appendix 2.

#### Engagement Efficiency

To measure the app’s engagement efficiency, an analysis of objections raised by users was performed. It is important for a real-world conversational app to understand users’ written messages with high accuracy, precision, and recall to provide empathetic listening and to correctly interpret and respond to a user every single time. This is critical to provide seamless user engagement and experience, which in turn leads to higher app usage and retention. All the conversation messages (instances) the users had with the app were manually tagged for “objection” or “no objection.” Objections took 2 forms: refusals (ie, when the user objects to a bot’s understanding of what was said; for eg, “I don’t want to do this”) and complaints (ie, when the user raises a complaint to a bot’s response; for eg, “That’s not what I said”). See Multimedia Appendix 7 for examples on objections. The proportion of objections raised by a user was measured for prevalence. The tagged dataset was also used to evaluate the performance of an existing supervised ML classifier algorithm deployed to automatically detect objections in real-world use. For details about this analysis, see Multimedia Appendix 2 *.*

## Results

### Analysis Size

The mixed-methods analysis was performed on 129 users (high users, n_h_=108; low users, n_l_=21) who had met the inclusion criteria.

### Quantitative Analysis

#### Impact (Pre-Post) Analysis

The study first screened for users who self-reported a Pre-PHQ-2 score equal to 6. We initially checked that users’ PHQ-9 scores had improved (ie, reduced going from pre- to post), on average, between time points. Both comparison groups showed a significant reduction in PHQ-9 score (within groups) as measured by a Wilcoxon signed-rank test (Table 1). The authors expected that regression to the mean (whereby values that are initially measured as extreme are more likely to be moderate on subsequent measurement) might play a role in this apparent large improvement [40].

Therefore, a between-groups comparison of the average improvement (Pre-PHQ-9 minus Post-PHQ-9) was performed using a Mann-Whitney *U* test (Table 2). We found that the high users group showed significantly higher average improvement compared with the low users group (*P*=.03). The effect size was found to be approximately 0.63. For the purposes of post hoc comparisons, other studies have found that a CL of 0.63 is roughly equivalent to a Cohen *d* of 0.47 [39]. For quality control purposes, as discussed in the paper by Zimmerman [41], an unpaired *t* test with outliers removed was then conducted. This also produced a significant result (*P*=.028).

As a post hoc analysis, the PHQ-2 screening cutoff score was reduced so that additional Wysa users could be added to the sample. With a PHQ-2 cutoff score of 5, the high users group still showed higher average improvement compared with the low users group, but the effect was less significant (*P*=.06). With a PHQ-2 cutoff score of 4, the same effect was observed but at an even lower significance (*P*=.09).

#### Context/Descriptive Analysis

In total, 83.3% (90/108) of high users actively used the app for more than 4 days on and between 2 consecutive PHQ-9 screenings (see Multimedia Appendix 8). Given the natural app-use environment, each user in both groups had different pre- and postscreening days that were spaced at least 2 weeks apart within the study period.

**Table 1:** Within-group analysis.

Users with self-reported PHQ^a^-2=6		Number of users (N)	Mean (scores)	Median (scores)	W-value (*P* value^b^)
**High users**					
	Pre-PHQ-9	108	18.92	19.50	478.5 *(P*<.001)
	Post-PHQ-9	108	13.07	12.00	—
**Low users**					
	Pre-PHQ-9	21	19.86	21.00	32.5 (*P*=.01)
	Post-PHQ-9	21	16.33	17.00	—

^a^PHQ: Patient Health Questionnaire.

^b^95% significance.

**Table 2:** Between-group analysis.

Users with self-reported PHQ-2^a^=6	Number of users (N)	Mean improvement (SD)	Median improvement	Mann-Whitney *U* (*P* value^c^)	Effect size (CL^b^)
High users (n_h_)	108	5.84 (6.66)	6.00	835.5 (*P*=.03)	0.632
Low users (n_l_)	21	3.52 (6.15)	2.00	—	—

^a^PHQ-2: Patient Health Questionnaire-2.

^b^CL: common language effect size.

^c^95% significance.

In addition, 80.6% (104/129) of users gave a postscreening within 18 days of a prescreening (see Multimedia Appendix 9). The users came from diverse time zones (see Multimedia Appendix 10); 48.1% (62/129) of users came from America, followed by 26.4% (34/129) from Europe and 18.6% (24/129) from Asia. A total of 89.9% (116/129) users reported a recent major event or change in their life (see Multimedia Appendix 11). A total of 26.7% (31/116) cited “relationship issues/changes” as a recent major event. Among relationship issue/change, “break-up” was the top cited issue (11 of the 31), followed by “concerns and challenges with close family member” (8 of the 31). Other relationship issues or changes included issues with friends (3 of the 31), issues with other relations (3 of the 31), conflicts in marriage (3 of the 31), and getting into a new relation (3 of the 31). A total of 12.9% users (15/116) reported “mental well-being changes” as a recent event. Moreover, 5 of the 15 acknowledged they had multiple well-being issues, and 4 of the 15 acknowledged going through depression. In addition, 10.3% (12/116) mentioned “change of location” and 9.5% (11/116) mentioned facing a “personal loss or bereavement.” Furthermore, 90.7% (117/129) of users reported “hard to cope” or “slightly hard to cope” (see Multimedia Appendix 12), signifying a high percentage of users giving themselves a negative self-rating on their current ability to cope with daily tasks. A total of 59.7% (77/129) of users assessed and completed at least 1 wellness tool provided by the app (see Multimedia Appendix 13). Among those who completed, 72 were high users and 5 were low users. The remaining 40.3% (52/129) who did not complete a wellness tool only conversed with the app and likely assessed a wellness tool but not complete it. For details on most frequently reported major events or changes by 2 or more users, see Multimedia Appendix 14. The authors recognize that there would be overlap among the defined major event categories, which was a challenge to address given the anonymity of the users.

### Qualitative Analysis

#### Engagement Effectiveness

In all, 73.6% (95/129) of users provided at least one response to the in-app feedback questions. Of those who responded, 86 were from the high users group and 9 were from the low users group. A total of 282 feedback responses were received from these 95 users. In total, 60.9% (172/282) responses were received for the in-app question “Have I been able to help you feel better yet?” that was asked at the end of each user session. A total of 90.8% (256/282) semistructured responses were received by choosing app-provided preformatted options. The remaining 9.2% (26/282) responses were by way of free-text and were provided by 17 of the 129 users.

**Figure 2:**
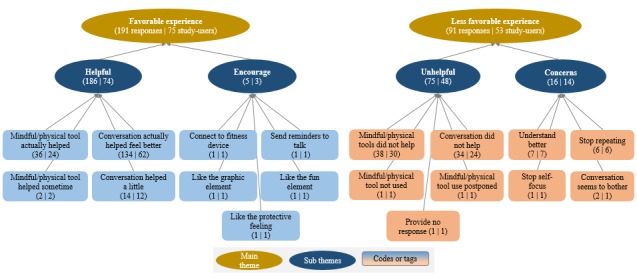
Thematic map with prevalence.

Thematic analysis was carried out on the 282 responses received from the users. Two main themes emerged, one “Favorable Experience” with the subthemes Helpful and Encourage and the other “Less Favorable Experience” with the subthemes Unhelpful and Concerns. The thematic map with prevalence can be seen in Figure 2 *.* A total of 67.7% (191/282) responses provided by 75 users found the app experience favorable. Of those favorable, 97.4% (186/191) responses found the conversation with the app and the tools helpful. A total of 32% (91/282) responses provided by 53 users found the app experience less favorable. Of those less favorable, 82% (75/91) responses found the conversation and tools either not helpful or did not use the tools; 13 responses (14%, 13/91) pointed to the app as not understanding or repeating, and a small fraction of 3 responses (3%, 3/91) mentioned that the app was self-focused and conversations seemed to bother the user.

Only 17 of the 129 users provided free-text feedback responses that provided additional insight into users’ in-app experience. The free-text responses were analyzed keeping in perspective the user context as identified in the Context/Descriptive Analysis subsection within the Quantitative Analysis Results section. For a detailed analysis of the free-text in-app feedback responses, see Multimedia Appendix 15.

Favorable experience was the dominant theme from the user responses*.* Almost all of the favorable experiences were attributed to the helpfulness of the app in users actually feeling better after their conversation sessions and also after their use of app-provided mindfulness and physical activity techniques. Users mostly chose the preformatted response option of “Yes, Actually” in response to the feedback question “Have I been able to help you feel better yet?” that acknowledged that the app conversation and mindfulness or physical activity techniques were actually helping them feel better. If users found app-based conversations or mindfulness and physical activity techniques not helpful or expressed any concern, it was classified as a less favorable experience. Among those who provided a less favorable experience, 2 users postponed use or did not use the techniques or tools during the study period. These were also considered as a less favorable experience given that the users were not motivated enough to try out the techniques or tools. Users mostly chose the preformatted response option of “Not, Really” or “Not yet” in response to the feedback question “Have I been able to help you feel better yet?” that acknowledged that the app conversation and mindfulness or physical activity techniques did not help the user feel better. Some users chose the preformatted option of “Understand me better” or “Too repetitive” in response to in-app feedback question “Anything specific you’d like to improve?”

**Figure 3:**
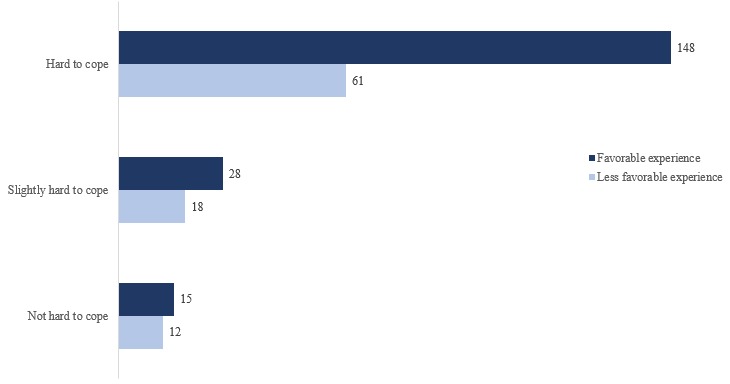
Coping experience–based feedback response distribution.

Of the 95 users who provided the 282 responses, those who reported hard to cope with daily tasks reported a higher proportion of favorable experience responses compared with less favorable experience responses (Figure 3).

**Figure 4:**
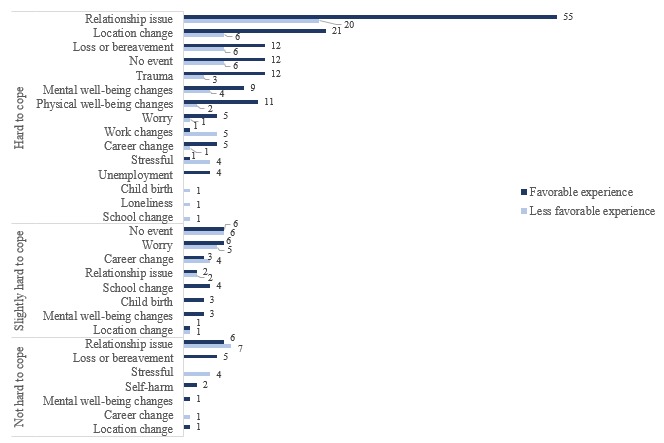
Coping major event-app experience-based feedback response distribution.

Among those who reported hard to cope, those who reported relationship issues or changes as a major event expressed a significantly higher proportion of favorable experience responses compared with less favorable experience responses (Figure 4). Those who did not face coping challenges were mostly found to be mixed about their experience with the app.

#### Engagement Efficiency

A total of 8075 anonymized conversational instances were obtained from 129 users during the study period. A relatively small proportion, 1.58% (128/8075) instances, of objections were observed in the conversation with the app.

The existing supervised classification-based ML algorithm that was deployed to classify objections in real time was tested on these 6611 instances. The remaining 18.13% (1464/8075) instances were ignored by the algorithm as the messages contained emoticons, texts in multiple lines, and special characters. The classifier model provided the following performance:

- Accuracy: 99.2% of objections and no objections that was detected was actually correct
- Specificity: 99.7% of no objections that was detected was actually correct
- Precision: 74.7% of objections detected (classified) was actually correct
- Recall: 62.1% of actual objections was detected (classified) correctly

**Figure 5:**
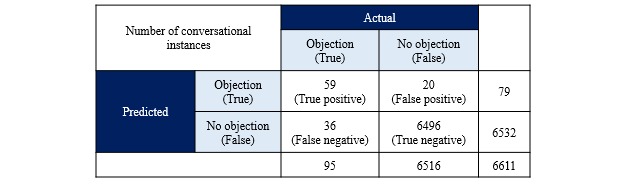
Confusion matrix of the objection handling machine learning model.

See Figure 5 for the confusion matrix.

## Discussion

### Principal Findings

The study revealed that the high users group had a significantly higher average improvement score in self-reported symptoms of depression compared with the low users group at a stringent PHQ-2 cutoff.

We found a significant reduction in PHQ-9 scores in high users and low users groups. We attribute the latter to the regression to the mean, suspecting that regression to the mean also plays a role in the high users group. Although the comparison group of “low users” does not fully constitute a control group, it provided an attempt to account for regression to the mean, as the reduction in PHQ-9 score seen in the high users group was significantly greater than that of the low users group. Users in both groups used the app during the full study period; therefore, they had comparable expectations that possibly reduced some biases.

A less significant effect was observed when the stringent cutoff PHQ-2 score was reduced. One explanation is that the app is most effective for people who show more severe symptoms of depression. As this is an in-the-wild study with no face-to-face screening, it is likely that lowering the PHQ-2 threshold score increased the number of people in the sample who were not mentally unwell and thus introduced additional unaccounted-for variability. Future work should deploy repeated measure questionnaires such as Resilience Scale RS-14 [42], which may be more sensitive to changes in resilience in the general population.

Relationship issues, mental well-being issues, location change, loss or bereavement, and career change formed the top major events or changes reported by users. Breakups and challenges with family members were the most common relationship issues. A recent study [43] has found that good mental health is not only the absence of symptoms but also what the user rates about his or her current ability to cope. Individuals who rated their current mental health as good had 30% lower probability of having a mental health problem at follow-up. Given the high proportion of negative self-rating on ability to cope in this study, the average improvement in self-reported symptoms of depression among high app users in a relatively short time period appears promising.

A high percentage of our study users (74%) provided in-app feedback. Most preferred to respond by clicking preformatted options presented by the app rather than free-text. A higher proportion of feedback found the app helpful and encouraging. There was an almost equal proportion of users who found the mindfulness and physical activity tools and techniques both helpful and not helpful, suggesting mixed experiences. Some suggested improvements included wanting the app to understand them better and wanting to avoid repetitions. Users who expressed hard to cope with daily tasks and who reported facing relationship issues in the recent past found the app helpful and gave a higher favorable experience feedback.

User objections (refusals or complaints) formed a relatively small proportion (1.58%). The existing objection detection ML model gave higher values for accuracy but lower for recall and precision, suggesting a need for further tuning of the model to reduce false positives and false negatives. A high performing ML model would become a necessity when conversation volumes increase to ensure high user engagement and retention. Continuous measurement of the objection rate can help provide an internal benchmark for chatbot apps to improve upon their engagement efficiency.

### Comparison With Prior Work

Our study results were compared with other RCT studies [31,32] using an automated text-based conversational agent intervention to study impact on participants’ mental well-being. One feasibility study (“first study”) compared reduction in symptoms of depression from 2-week use of a CBT-oriented instant messenger-based conversational agent against an information control group in a nonclinical college population (n=70) [31]. The other pilot study (“second study”) compared increased levels of psychological well-being from 2-week use of a positive psychology-oriented smartphone-based conversational agent against a wait-list control group in a nonclinical population (n=28) [32]. Both the studies reported between-group effect sizes based on the parametric Cohen *d*. The first study used PHQ-9 reporting a medium effect size of *d*=0.44 (from intent-to-treat analysis). The second study used the Flourishing Scale, Perceived Stress Scale, and Satisfaction with Life Scale and reported an effect size range of *d*=0.01 to 0.91 (from intent-to-treat analysis). The equivalent Cohen *d* of 0.47 (for CL of 0.63) from our study was comparable with that reported from the first study.

Both studies processed qualitative data gathered from responses to open-ended questions at postmeasurement using thematic analysis (Braun and Clarke, 2006). Although the approach taken differed from our study, there were similarities in observed experiences. The proportion of favorable responses (58 of 89 participants; 65%) to less favorable responses (31 of 89 participants; 35%) in the first study was similar to our study (68%:32%), suggesting users in both the studies reported a similar experience with a chatbot app. This observation will need validation in future studies. Users in our study and the first study highlighted the helpfulness of the conversation and the encouragement received, along with the feedback that chatbot provided an element of fun. Among the less favorable experiences, users (our study and first study) pointed to the repetitiveness of the conversation and a need for the app to understand the user better.

We also compared between-group effect sizes from 2 other RCTs that compared a Web-based human therapy intervention for depression with a waiting list [44,45]. We observed that our study effect size fell within the range of effect sizes reported (0.18-0.81) in those studies and closer to the larger effect size at follow-up. Our study effect size was also compared with the effect sizes reported in a 2018 meta-analysis [22] of RCT studies published before September 2016. The effect size from the 32 studies on major depressive disorders was found to range between 0.51 and 0.81 (Hedges *g*). Our study effect size was close to this effect range.

There are no known published metrics to compare how the “Objection Rate” fares among chatbot users with self-reported symptoms of depression. The observed objection rate of 1.58% when compared with the overall objection rate of 0.83% when all app users during the study period were considered (including excluded users); it is seen that the objection rate of users with self-reported symptoms of major depression (PHQ-2=6) was higher. This might indicate that users with high self-reported symptoms tend to object more during their conversation with a well-being app. Extensive research is needed in this area, especially given the ethical issues that may arise.

### Value of the Study

The study design allows for scalability to conduct large longitudinal studies and, therefore, a relatively easier and early assessment of a chatbot’s real-world effectiveness and engagement. The in-app based feedback approach allowed for real-time insights into the users’ experience using a personalized intervention, without the danger of losing vital feedback and insights due to delays in collection. The study outlines a way to use existing conversational inputs to gather additional context about the user when no personally identifiable information or demographic information is collected. This is an approach that will aid in personalizing the user experience when conversing with a chatbot app. There exists tremendous value and potential for the app to enable Ecological Momentary Assessment (EMA) or Experience Sampling Method (ESM). Our study team supports the adoption of EMA or ESM as a research method for future studies where the objectives involve a more intensive, repeated, and momentary capture to assess changes in behavior, emotions, and mood of users. In future longitudinal studies, it will also add value to report on important app engagement measures such as user retention to complement the study findings. In a real-world context as conversations scale, the study recommends a need to evaluate and build high-performing ML models, including evaluation of unsupervised learning approaches, to detect objections in real-time while ensuring better control and interpretability of the model results. This allows for early handling of user objections to help make the chatbot more empathetic, enhance user engagement and retention, and strive for high ethical standards.

### Limitations of the Study

A study of this nature has a number of limitations. A lack of a randomized controlled environment would lead to nonhandling of biases. No prior health information exists about the users, particularly their past or ongoing clinical history, diagnosis or treatment, or presence of comorbidities that could impact the effect. Both PHQ-2 and PHQ-9 have good acceptability for screening but do not confirm clinical diagnosis of depression (ie, participants with high PHQ-9 scores need not necessarily have depression and vice versa). This study design is a form of quasi-experimental design and is slightly lower in design quality compared with interrupted time-series designs (multiple pretest and posttest observations spaced at equal intervals of time). Statistical limitations include small and unbalanced comparison group sizes and not being able to account for variables such as age, gender, or socioeconomic status. A lack of detailed feedback responses on users’ app experience limits the data available to gain insights using a qualitative analysis.

Bias may also exist in the form of increased exposure to certain features in the app for the high users group, which may partly contribute to influencing users in unknown ways. There is a need to insulate the app’s design (such as color themes, font types, text alignments, icons, and emoticons) from contributing to the effects observed. The study sample size was too small to examine how people reacted to the app design elements and how that impacts their symptoms of depression. The authors intend to further delineate these issues in future research with larger samples.

Handling of these limitations would be a subject for future studies including the conduct of more elaborate comparison studies.

### Conclusions

Our study identified a significantly higher average improvement in symptoms of major depression and a higher proportion of positive in-app experiences among high Wysa users compared with low Wysa users. These findings are encouraging and will help in designing future studies with larger samples and more longitudinal data points.

## Appendix

Multimedia Appendix 1 Wysa app study version.

Multimedia Appendix 2 Supplementary methods.

Multimedia Appendix 3 Mixed methods approach followed for the study.

Multimedia Appendix 4 Study recruitment chart. "Enrollment" depicts inclusion of users who provided only 2 valid PHQ-9 assessment (pre and post over 14 days apart). “Allocation” splits users into 2 comparison groups based on their app usage on and between the two screening time-points. “Analysis” includes users who scored a total of “6” in the first 2 items of their PHQ-9.

Multimedia Appendix 5 In-app feedback question and responses.

Multimedia Appendix 6 A typical user engagement with the Wysa app. Time period 1 denotes the start of app use by a user. Time period n denotes the end of the study period.

Multimedia Appendix 7 Sample objection quotes from users.

Multimedia Appendix 8 Distribution of users based on Total Active Days on and between screening days.

Multimedia Appendix 9 Distribution of Wysa app users based on number of days between the screening days.

Multimedia Appendix 10 Region and time-zones of all included users.

Multimedia Appendix 11 Distribution of self-reported major events and changes of all included users.

Multimedia Appendix 12 Distribution of self-reported ability to cope among all included users.

Multimedia Appendix 13 Distribution of users who completed wellness tools.

Multimedia Appendix 14 Major events or changes reported by users.

Multimedia Appendix 15 Analysis of free-text in-app feedback responses from study users.

## Abbreviations

AI: artificial intelligence
CBT: cognitive behavioral therapy
CL: common language effect size
EMA: Ecological Momentary Assessment
ESM: Experience Sampling Method
ML: machine learning
NHS: National Health Service
PHQ-2: 2-item Patient Health Questionnaire
PHQ-9: 9-item Patient Health Questionnaire
RCT: randomized controlled trial
WHO: World Health Organization
