# Supplementary figures and images for: An Empathy-Driven, Conversational Artificial Intelligence Agent (Wysa) for Digital Mental Well-Being: Real-World Data Evaluation Mixed-Methods Study
Source: JMIR Mhealth Uhealth. 2018 Nov 23;6(11):e12106. doi: 10.2196/12106 (PMC6286427; doi:10.2196/12106)

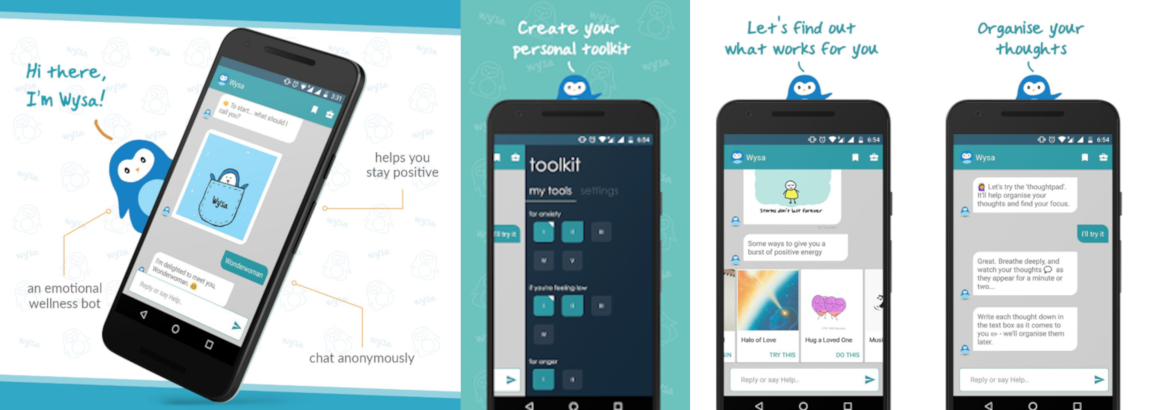

Supplement: Multimedia Appendix 1 [file mhealth_v6i11e12106_app1.png]

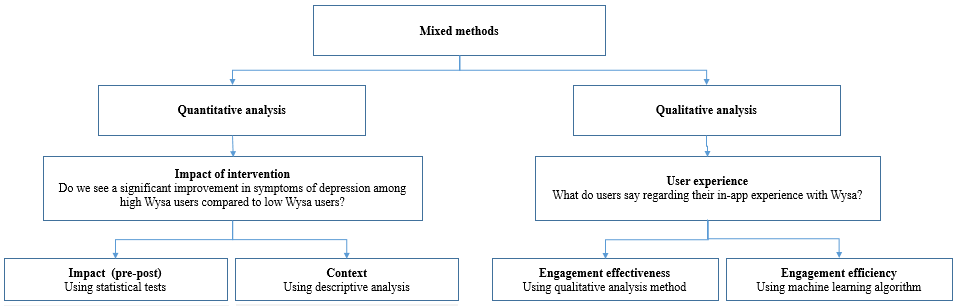

Supplement: Multimedia Appendix 3 [file mhealth_v6i11e12106_app3.png]

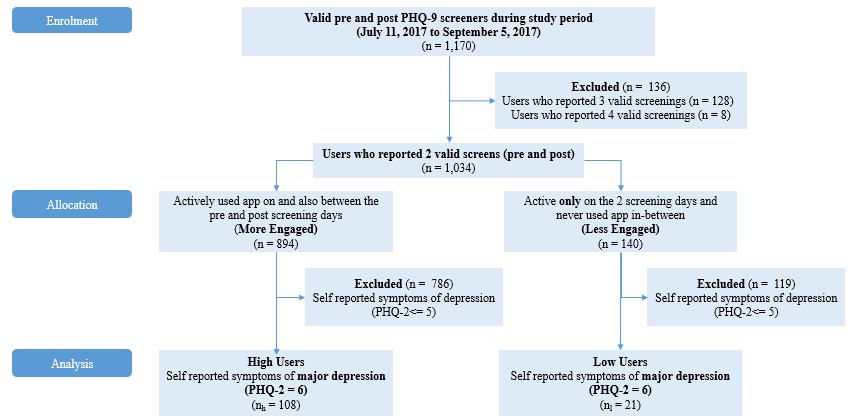

Supplement: Multimedia Appendix 4 [file mhealth_v6i11e12106_app4.png]

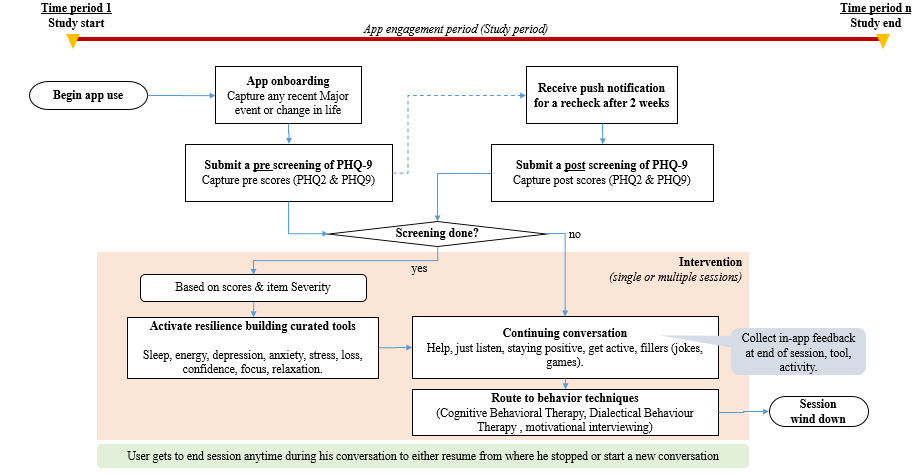

Supplement: Multimedia Appendix 6 [file mhealth_v6i11e12106_app6.png]

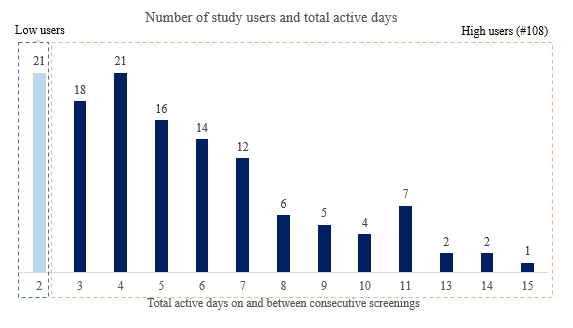

Supplement: Multimedia Appendix 8 [file mhealth_v6i11e12106_app8.png]

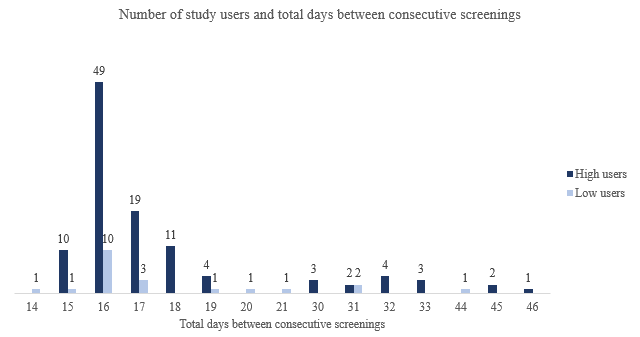

Supplement: Multimedia Appendix 9 [file mhealth_v6i11e12106_app9.png]

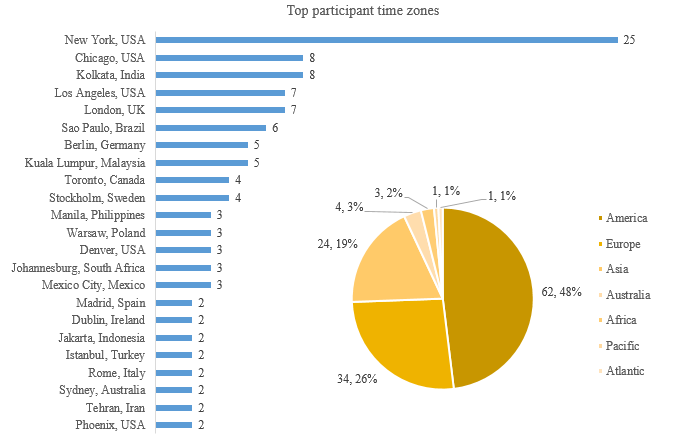

Supplement: Multimedia Appendix 10 [file mhealth_v6i11e12106_app10.png]

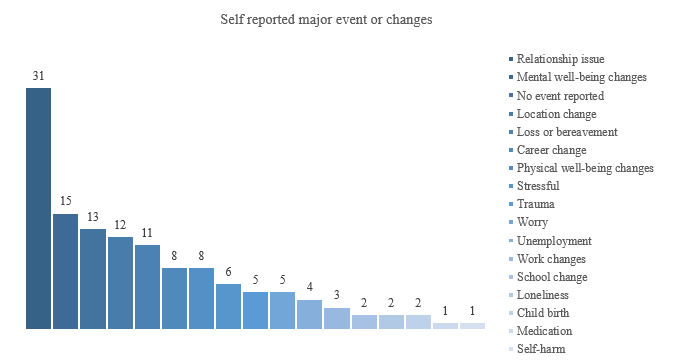

Supplement: Multimedia Appendix 11 [file mhealth_v6i11e12106_app11.png]

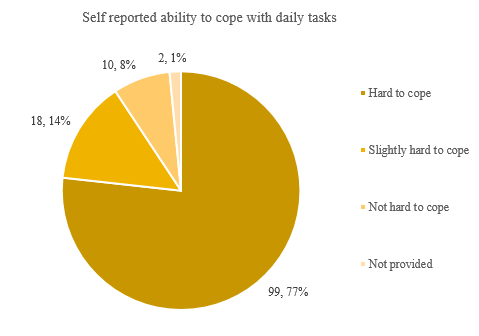

Supplement: Multimedia Appendix 12 [file mhealth_v6i11e12106_app12.png]

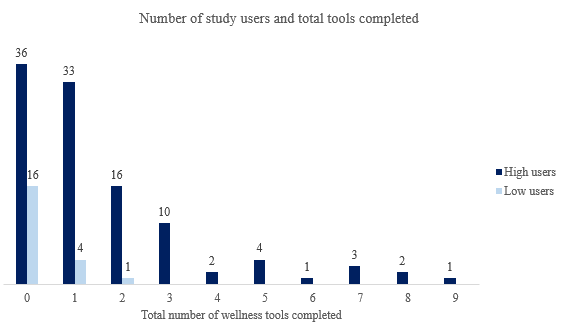

Supplement: Multimedia Appendix 13 [file mhealth_v6i11e12106_app13.png]
