# Supplementary material for: An Empathy-Driven, Conversational Artificial Intelligence Agent (Wysa) for Digital Mental Well-Being: Real-World Data Evaluation Mixed-Methods Study
Source: JMIR Mhealth Uhealth. 2018 Nov 23;6(11):e12106. doi: 10.2196/12106 (PMC6286427; doi:10.2196/12106)
Supplement: Multimedia Appendix 2 [file mhealth_v6i11e12106_app2.pdf]

Multimedia Appendix 2: Supplementary methods

App-Specific Ethical Practices

The Wysa app is not a medical device and not intended to replace and provide therapy for a condition or disease. Anonymity is a key design principle within the Wysa app as it meant to promote user trust and engagement, including higher privacy and data protection. The app design and development decisions is governed by Privacy-by-Design principles and the Standardisation Committee for Care Information standards for clinical safety namely; SCCI0129 and SCCI0160. All in-app conversational text, modules, tools and techniques used by the study app is evidence-based. For example, apart from using established and validated Cognitive Behavioural Therapy (CBT) techniques, each tool that was customized for the user included an available reference to an external peer-reviewed scientific paper along with a lay summary with suggested benefits articulated by the referenced paper. The user voluntarily took the PHQ-9 at the time of on-boarding and after every two weeks. The total scores were not directly made available to the users; instead, based on the scores, validated empathetic feedback messages were provided. An example of feedback message provided based on self-reported scores and ability to cope with daily tasks would include; "You seem to be doing well overall, but are struggling with a few problems right now. However do consider talking to a psychologist about strategies that work for others" or "It seems you are experiencing some difficulty with keeping up a positive emotional state. However do consider talking to a psychologist about strategies that work for others."

Mixed Methods Design and Approach

A concurrent triangulation mixed methods design [1] was explored to compare and contrast the quantitative and qualitative results and help corroborate findings. Based on this design, the two forms of data were analysed separately but concurrently and later combined to provide a comprehensive view. **Multimedia Appendix 3** outlines the mixed method approach that was followed, where both quantitative and qualitative data were analysed to answer the questions recorded under the study objectives.

About PHQ-9

The PHQ-9 generated scores is associated with each of the nine Diagnostic and Statistical Manual of Mental Disorders 4th edition (DSM-IV) criteria as 0 (not at all) to 3 (nearly every day). It provided an overall score (from 0 to 27) that could be measured over time to monitor improvement or worsening of symptoms of depression [2].

Thematic Analysis Approach

The thematic method, Braun & Clarke (2006), involved a mix of both inductive and theoretical analyses with an emphasis on a 'bottom up' exploratory approach (inductive). Each in-app feedback response instance was reviewed and tagged with an initial code that captured the user's context. Instances tagged to similar codes were then grouped and reviewed again to ensure that the initial codes were an accurate reflection of all the grouped instances. The final set of codes and their grouped instances were reviewed and sorted to identify potential subthemes or themes. The initial thematic map was then constructed. The instances within the potential sub-themes and themes were reviewed once again to ensure they cohered together meaningfully and that each theme was clear and distinct. The theme names were then finalized and the final thematic map was constructed.

Engagement Efficiency Analysis

The analysis was performed in five stages:

1. **Objective stage:** The objective of the analysis was to detect the extent of objections raised by the users and to explore the performance of a proprietary ML algorithm to automatically detect objections in real-time.
2. **Data gathering stage:** All the text-based conversation data received from the users were securely extracted and de-identified for app identifiers prior to analysis.
3. **Data preparation stage:** Each user conversation instance was reviewed manually for objections and labelled as 1 for “objection” and 0 for “no objection”. For ML modelling, feature extraction and Term Frequency–Inverse Document Frequency (TF-IDF) transformation was used to vectorise each labelled conversation instance. Unnecessary and redundant words were removed. Lastly, Feature Engineering was performed where POS (Parts of speech) tagging was completed followed by extraction of bi-grams from the POS tagged corpus. The transformed dataset was split into training (90%) and test (10%).
4. **Data modeling stage:** The ML model was used to fit, transform and classify the transformed dataset. A Stochastic Gradient Descent (SGD) Classifier algorithm was used for classification modelling. A k-fold cross validation (K=10) was performed on the training set and the models were evaluated using the f1-score (weighted average of precision and recall). The best model was trained on the entire training set before the model performance was externally validated on the unseen test dataset.
5. **Interpretation stage:** To measure prevalence, the proportion of objections raised by the users was derived. To measure the ML model performance, the model accuracy, recall and precision values were measured. A high accuracy was important to verify what proportion of classification the model correctly predicted. Recall and precision were also measured given that the model needed to capture lower false positives and false negatives. Recall answers the question “What proportion of actual objections was detected (classified) correctly?” whereas precision answers the question “What proportion of objections detected was actually correct?”

References

1. Creswell JW, Plano CVL, Gutmann ML, Hanson WE. Advanced mixed methods research designs. In A.Tashakkori & C. Teddlie (Eds.). Handbook of mixed methods in social and behavioral research 2003:209-240. Thousand Oaks, CA: Sage Publications. ISBN:1412972663
2. Kroenke K, Spitzer RL, Williams JBW. The PHQ-9: Validity of a brief depression severity measure. J Gen Intern Med 2001; 16(9): 606–613. doi:10.1046/j.1525-1497.2001.016009606.x
