# Supplementary material for: An Empathy-Driven, Conversational Artificial Intelligence Agent (Wysa) for Digital Mental Well-Being: Real-World Data Evaluation Mixed-Methods Study
Source: JMIR Mhealth Uhealth. 2018 Nov 23;6(11):e12106. doi: 10.2196/12106 (PMC6286427; doi:10.2196/12106)
Supplement: Multimedia Appendix 5 [file mhealth_v6i11e12106_app5.pdf]

Multimedia Appendix 5: In-app feedback question and responses

Reference	Feedback question	Details	Pre formatted response options	Total responses
Q1	"Have I been able to help you feel better yet?"	**Objective:** Obtain Session effectiveness<br>**Frequency:** At the end of every wellness based session<br>**Trigger question:** When user responds positively to bot-asked question **"Before you go can I ask how I'm doing so far?"**	"Yes, actually";<br>"Not Yet";<br><br>Trigger options<br>"I like it"; (positive)<br>"Not today";<br>"So Far, So Good"; (positive)<br>"Not Very Well"; (negative)	172
Q2	"Did that help?"	**Objective:** Obtain mindfulness technique effectiveness<br>**Frequency:** At the end of every mindfulness session	"Listen to me";<br>"Yeah actually";<br>"Not Really";	63
Q3	"Do you feel better?"	**Objective:** Obtain Physical activity technique effectiveness<br>**Frequency:** At the end of every tool-based session	"Yes, Actually";<br>"Not Really";	19
Q4	"Anything specific you'd like to improve?"	**Objective:** Obtain Session effectiveness<br>**Frequency:** At the end of every wellness based session<br>**Trigger question:** When user responds negatively to bot-asked question **"Before you go can I ask how I'm doing so far?"**	" Too Repetitive";<br>"No";<br>"Understand me Better"<br><br>Trigger options<br>"I like it"; (positive)<br>"Not today";<br>"So Far, So Good"; (positive)<br>"Not Very Well"; (negative)	15
Q5	"Is the conversation helping you?"	**Objective:** Obtain Loss-based session effectiveness<br>**Frequency:** At the end of every loss-based session	"Yes Quite";<br>"A Little";<br>"Not Really";	11
Q6	"I'm still very new,	**Objective:** Obtain feedback on improvement areas	Only Free Text Entry	1

Reference	Feedback question	Details	Pre formatted response options	Total responses
	and your feedback helps me grow. What do you think I could do better?”	**Frequency:** Every weekend		
Q7	“Anything else I could do better?”	**Area:** Obtain feedback on improvement areas<br>**Frequency:** Every weekend	Only Free Text Entry	1
			**Total**	**282**
