# Supplementary material for: An Empathy-Driven, Conversational Artificial Intelligence Agent (Wysa) for Digital Mental Well-Being: Real-World Data Evaluation Mixed-Methods Study
Source: JMIR Mhealth Uhealth. 2018 Nov 23;6(11):e12106. doi: 10.2196/12106 (PMC6286427; doi:10.2196/12106)
Supplement: Multimedia Appendix 7 [file mhealth_v6i11e12106_app7.pdf]

Multimedia Appendix 7: Sample objection quotes from users

Objection quotes
“Can we talk about something else?”
“I don't understand”
“I just told you”
“I don't want to.”
“I don't need this now”
“No. Give a solution”
“I'm not going to”
“This is not a good time to talk”
“I don't wanna talk about it.”
“You have already asked that, bot~”
“Sorry you're not helping me”
“You don't help”
“you just sound ignorant that's all”
“Stop and listen to me let me speak”
“Doesn't work for now”
“Stop this session”
“Stop the mindreading”
“Stop repeating yourself”
“This is not working”
“Can you rephrase that?”
“You do not understand me!!!"
“They can't help me!”
