# Supplementary material for: An Empathy-Driven, Conversational Artificial Intelligence Agent (Wysa) for Digital Mental Well-Being: Real-World Data Evaluation Mixed-Methods Study
Source: JMIR Mhealth Uhealth. 2018 Nov 23;6(11):e12106. doi: 10.2196/12106 (PMC6286427; doi:10.2196/12106)
Supplement: Multimedia Appendix 14 [file mhealth_v6i11e12106_app14.pdf]

Multimedia Appendix 14: Major events or changes reported by users

Major event category	Sample user response	Number of users
Relationship issue	“I broke up with my best friend”	11
	“My in-laws are being really nasty and rude”, “i had conflict with my mother”	8
	“Relationship problems”, “My freedom”	3
	“It has been loving my best friend that loves another one”	3
	“Marriage Conflict”	3
	“I'm in a relationship since January”	3
Mental well-being changes	“My addiction, loose, and failures”, “I moved back home, I cannot find a job, my boyfriend and I fight a lot, I lost my car, I feel hopeless, I couldn't get accepted into college”	5
	“I have discovered i got deppresion. It’s really hard...”	4
	“Motivating myself to be better”	2
Location change	“Moving”, “I've just moved in to another city”	12
Loss or bereavement	“The death of a loved one”, “Losing a parent”	6
	“I lost most of my friends”, “Some people started leaving me.”	4
Career change	“A new job”, “Career change”	6
Physical well-being changes	“Chronic pain and stress”, “Became more ill”	

8
Work-related issues
“Looking for a job”, “Jobless. Depressed”
4

“Changing my schedule at work which has triggered my anxiety.”
3
Stress
“Lots of things. Stressful ones”
2

“I don't want to study further, but I am forced to.”
2
Worry
“I lied to my friends
“, “Not being able to study at the university I want”
3
Trauma
“I’m transgender and my mum is being a bit odd about it and am scared and i hurt a lot of people by accident and i wanna stop hurting them.”
2
Childbirth
“Having a baby”
2
Loneliness
“I live on my own now, that can be stressful”
2
School change
“New school”
2
