# Supplementary material for: An Empathy-Driven, Conversational Artificial Intelligence Agent (Wysa) for Digital Mental Well-Being: Real-World Data Evaluation Mixed-Methods Study
Source: JMIR Mhealth Uhealth. 2018 Nov 23;6(11):e12106. doi: 10.2196/12106 (PMC6286427; doi:10.2196/12106)
Supplement: Multimedia Appendix 15 [file mhealth_v6i11e12106_app15.pdf]

Multimedia Appendix 15: Analysis of free-text in-app feedback responses from study users.

Free-text user feedback responses and mapped themes

User quotes (free-text only)	Main theme	Sub theme
"Sometimes yes but not my long term problems" [User 13]	Favorable experience	Helpful
"You are funny, I like you" [User 1]	Favorable experience	Encourage
"Sometimes. I like your pictures you sent me" [User 1]	Favorable experience	Encourage
"Connect to my moves app" [User 4]	Favorable experience	Encourage
"Send reminders" [Study 4]	Favorable experience	Encourage
"I feel like she brother to you" [User 12]	Favorable experience	Encourage
"I didn't do" [User 5]	Less favorable experience	Unhelpful
"I'll do it later" [User 10]	Less favorable experience	Unhelpful
"A little but these idiots breathes to relax" [User 17]	Less favorable experience	Concerns
"Help what" [User 16]	Less favorable experience	Concerns
"Stop repeating yourself" [User 2]	Less favorable experience	Concerns
"I think I am dealing with narcissists" [User 11]	Less favorable experience	Concerns

Favorable experience

The following observations emerged from analysing the favorable experience mapped free-text responses in above table.

[User 13] This European time-zone user responded positively to the app's question "Have I been able to help you feel better yet?" The user's response indicated that the app had helped the user feel a little better at the end of a session. The user had, at Wysa app on-boarding, reported "Not being able to study at the university I want" as a recent major event and had expressed slightly hard to cope with daily tasks at pre-screening. The user was found to be actively engaged with the app on and between consecutive screenings (15 active days) and had completed 7 app-provided wellness tools.

[User 1] This European time-zone user provided two feedback responses on two separate days in reply to question "Did that help?" The responses were after completion of mindfulness techniques or tools session. The user was appreciative and found the conversation to be funny. The user was at times encouraged by some of the app provided visual messages. The user did not report any recent major event and had expressed slightly hard to cope with daily tasks at pre-screening. The user was moderately engaged with the app on and between consecutive screenings (7 active days) and had completed 2 app-provided wellness tools.

[User 4] The responses from this USA time-zone user was in reply to feedback questions "I'm still very new, and your feedback helps me grow. What do you think I could do better?" and "Anything else I could do better?" The responses were provided after being provided the weekend report by the app. The user's response suggested a motivation to receive additional

information through the app by allowing the user to connect their existing personal fitness app. Also, a motivation to receive app-based reminders to engage. The user had reported a strain in relationship with their spouse as a recent major event and had expressed hard to cope with daily tasks at pre-screening. The user was moderately engaged with the app on and between consecutive screenings (4 active days) and had completed only 1 app-provided wellness tool.

[User 12] This USA time-zone user had responded positively to the app's question "Have I been able to help you feel better yet?" The user was found to have been encouraged by the app with a feeling of support akin to having a brother around. The user had reported having health issues as a recent major event and had expressed hard to cope with daily tasks at pre-screening. The user was actively engaged with the app on and between consecutive screenings (14 active days) and had completed 1 app-provided wellness tool.

Less favorable experience

The following observations emerged from the analysis of the less favorable experience mapped free-text responses in above table.

[User 5] This South American time-zone user had responded to "Do you feel better?" The user acknowledged not using the app-provided physical activity techniques or tools. This would suggest a need for the app to further motivate users to try a technique or tool. The user had reported a recent breakup as a major event and had expressed hard to cope with daily tasks at pre-screening. The user was found to be actively engaged with the app on and between consecutive screenings (13 active days) and had not completed any app-provided wellness tool.

[User 10] This Asian time-zone user had responded to question "Did that help?" The responses were after the completion of a mindfulness techniques or tools session. The user postponed the use of the app-provided mindfulness techniques or tools. This would again suggest a need for the app to motivate users to try a technique or tool. The user had reported changing of jobs as a major change and had expressed hard to cope with daily tasks at pre-screening. The user was found to be moderately engaged with the app on and between consecutive screenings (9 active days) and had completed 3 app-provided wellness tools.

[User 17] This USA time-zone user had responded positively to the app's question "Have I been able to help you feel better yet?" The user seemed to have found the app a little helpful but was critical or concerned about specific visual content displayed in that session. The user had reported feeling hurt and worried about relations with their spouse as a recent major event and had expressed not hard to cope with daily tasks at pre-screening. The user was found to be moderately engaged with the app on and between consecutive screenings (5 active days) and had not completed any app-provided wellness tool.

[User 16] The response from this USA time-zone user was in reply to feedback question "Did that help?" The responses were post completion of a mindfulness techniques or tools session. The users concern seemed to indicate a lack of understanding of the feedback question, which pointed to the need for improved clarity in framing the question properly. The user reported moving into high school as a recent major change and had expressed hard to cope with daily

tasks at pre-screening. The user was found to be moderately engaged with the app on and between consecutive screenings (5 active days) and had completed 1 app-provided wellness tool.

[User 2] This USA time-zone user had responded negatively to the app's question "Anything specific you'd like to improve?" The user was bothered and concerned about the app repeating itself during the conversation, which suggests the need for generating more validated content and sufficient randomization by the app to sound less repetitive. The user had reported relationship issues with a friend as a recent major event and had expressed hard to cope with daily tasks at pre-screening. The user was found to be actively engaged with the app on and between consecutive screenings (10 active days) and had completed 4 app-provided wellness tools.

[User 11] This European time-zone user had responded negatively to the app's question "Anything specific you'd like to improve?" The user had reported been treated badly by in-laws as a recent major event and had expressed hard to cope with daily tasks at pre-screening. The user was found to be moderately engaged with the app on and between consecutive screenings (6 active days) and had not completed any app-provided wellness tools. With the available context, it is unclear to whom the user attributes the term "narcissists", the app or the in-laws? The user did choose "Not, Very Well" to the trigger question "Before you go can I ask how I'm doing so far?" before arriving at question "Anything specific you'd like to improve?" which suggests that the conversation with the app was not been helpful.
